# Lebensqualität bei Trägern eines suprapubischen oder transurethralen Harnblasenkatheters als lebenslange Dauerversorgung

**DOI:** 10.1007/s00120-021-01642-1

**Published:** 2021-10-04

**Authors:** A. Wiedemann, C. Gedding, M. Heese, J. Stein, A. Manseck, R. Kirschner‑Hermanns, H. Karstedt, A. Schorn, A. Wagner, V. Moll, U. Unger, A. Eisenhardt, A. Bannowsky, C. Linné, S. Wirz, E. Brammen, H.‑J. Heppner

**Affiliations:** 1 Urologische Abteilung, Evangelisches Krankenhaus Witten gGmbH, Lehrstuhl für Geriatrie, Universität Witten/Herdecke, Pferdebachstr. 27, 58455 Witten, Deutschland; 2 Lehrstuhl für Geriatrie, Universität Witten/Herdecke, Witten, Deutschland; 3 Urologische Abteilung, Klinikum Großburgwedel, Großburgwedel, Deutschland; 4 Urologische Abteilung, Klinikum Ingolstadt GmbH, Ingolstadt, Deutschland; 5 Neuro-Urologie, Universitätsklinikum Bonn, Bonn, Deutschland; 6 Neuro-Urologie, Neurologisches Rehabilitationszentrum Bonn-Godeshöhe, Bonn, Deutschland; 7 Praxis für Urologie, Gelsenkirchen, Deutschland; 8 Praxis für Urologie, Saarburg, Deutschland; 9 Praxis für Urologie, Limburgerhof, Deutschland; 10 Praxis für Urologie, Augsburg, Deutschland; 11 Praxis für Urologie, Oelsnitz, Deutschland; 12 Praxis für Urologie, Mülheim a. d. Ruhr, Deutschland; 13 Klinik für Urologie, Imland-Klinik Rendsburg, Rendsburg, Deutschland; 14 Urologische Praxis, Dresden, Deutschland; 15 Abteilung für Anästhesiologie, Intensivmedizin, Schmerz und Palliativmedizin, Zentrum für Schmerzmedizin, Weaningzentrum, GFO-Kliniken Bonn/Cura Bad Honnef, Bad Honnef, Deutschland; 16 Institut für Statistik, Chrestos Concept GmbH & Co. KG, Essen, Deutschland; 17 Geriatrische Abteilung und Tagesklinik, Helios-Klinikum Schwelm, Schwelm, Deutschland; 18 Institut für Biomedizin des Alterns, FAU Erlangen-Nürnberg, Erlangen, Deutschland

**Keywords:** Blasenfunktionsstörungen, Multimorbidität, Frailty, Geriatrischer Patient, Palliativversorgung, Bladder dysfunction, Multimorbidity, Frailty, Geriatric patient, Palliative care

## Abstract

**Hintergrund:**

Die Anlage eines transurethralen Dauerkatheters (DK) oder suprapubischen Harnblasenkatheters (SPK) in lebenslanger Indikation stellt einen Eingriff mit relevanten Komplikationen, Komorbiditäten und möglichen Auswirkungen auf die katheterassoziierte Lebensqualität des Betroffenen dar. Letztere wurde aber bisher noch nicht untersucht.

**Methodik:**

Zur Anwendung kam ein validiertes Assessment zur katheterbezogenen Lebensqualität mit 25 Items in 5 Domänen. Befragt wurden im Rahmen eines Katheterwechsels Patienten mit einem DK oder SPK in lebenslanger Intention, die diesen mindestens 3 Monate trugen.

**Ergebnisse:**

Fragebögen von 357 Patienten, davon 260 Männer und 97 Frauen, 193 mit SPK und 162 mit DK (2 ohne Angabe) lagen vor. Patienten mit DK waren mit 78,9 ± 11,1 Jahren signifikant älter als solche mit SPK mit 74,4 ± 12,6 Jahren (*p* < 0,001). Der mittlere Gesamtlebensqualitätsscore lag bei 4,1 ± 0,9 Punkten auf einer Skala von 1 (maximal beeinträchtigte Lebensqualität) bis 5 (keine Beeinträchtigung der Lebensqualität). Es zeigten sich u. a. mit niedrigeren Scores eine vermehrte Angst vor Katheterlecks, Angst vor Uringeruch und Harnwegsinfektionen und vor schmerzhaften Katheterwechseln. Diese Sorgen waren v. a. bei Frauen, solchen mit Harninkontinenz, Trägern eines Katheters ≥ 18 Ch und bei Patienten < 70 Jahren vorhanden. Frauen mit einem SPK wiesen eine schlechtere Bewertung ihrer Lebensqualität als Männer mit SPK auf.

**Schlussfolgerung:**

Die gefundenen Ergebnisse sollten in die Aufklärung zu einer lebenslangen Katheterableitung einfließen bzw. im Kontext möglicher Alternativen wie z. B. einer operativen Desobstruktion oder einer Hilfsmittelversorgung mit dem Patienten bzw. Betreuungspersonen besprochen werden.

**Zusatzmaterial online:**

Die Online-Version dieses Beitrags (10.1007/s00120-021-01642-1) enthält weitere Tabellen mit detaillierten Ergebnissen der Fragen der 5 abgefragten Domänen.

## Abstract (en)

**Background:**

The insertion of a Foley catheter (FC) or a suprapubic catheter (SPC) in lifelong intent is an intervention with significant complications, comorbidities and impact on the further life that has not yet been analyzed.

**Methodology:**

The analysis was based on a validated assessment of catheter-related QoL with 25 items in 5 domains and applied to patients with a Foley or suprapubic catheter in lifelong indication and with the catheter in place for at least 3 months. Assessment data were enriched with information on the type and diameter of the catheter as well as demographic data.

**Results:**

Questionnaires from 357 patients (260 male, 97 female, 193 with suprapubic catheter, 162 with Foley catheter, 2 no information) were included in the study. Patients with a Foley catheter were significantly older than patients with a suprapubic catheter (78.9 ± 11.1 years vs. 74.4. ± 12.6 years, *p* < 0.001). The average QoL score was 4.1 points on a scale from 1 (maximum impairment of QoL) to 5 (no impairment of QoL) indicating a moderately negative impact on QoL. Scores below the average were mainly driven and accompanied by a fear of urine leakage, urine odor, painful catheter changes and urinary infections increasing with age. Additionally, patients were worried about negative effects on their daily life activities due to the catheter. These worries seemed to be more pronounced in females with urinary incontinence, patients with a catheter size ≥ 18 Ch. and with an age of < 70 years. The type of catheter showed a greater impact on the QoL in females with suprapubic catheters when compared with males in contrast to patients with transurethral catheters.

**Conclusion:**

The results of the study provide further information for the medical clarification for patients and caregivers, having to decide between a lifelong catheter drainage or alternatives, such as provision of an aid or surgical recanalization.

Ob eine Blasenfunktionsstörung bei einem geriatrischen Patienten kausal oder palliativ mit einem Katheter behandelt wird, ist eine schwerwiegende und auch lebenslange Entscheidung. Die Erfolgsaussichten, Nebenwirkungen und Gefährdungspotentiale einer solchen kausalen Therapie müssen gegen die Komplikationen des Katheters und seine Auswirkungen auf die Lebensqualität abgewogen werden. Umso erstaunlicher ist es, dass der letzte Punkt systematisch noch nie untersucht wurde. Dies soll nun erstmalig mit einem validierten Assessment in dem vorliegenden Projekt des Arbeitskreises „geriatrische Urologie“ der DGU geschehen.

## Einleitung

Die Anlage eines transurethralen Harnblasendauerkatheters (DK) oder suprapubischen Katheters (SPK) in lebenslanger, palliativer Intention kann auch heute trotz vieler minimal-invasiver Methoden zur Beseitigung einer subvesikalen Obstruktion leitliniengerecht bei einer Harninkontinenz oder einer subvesikalen Obstruktion zur Anwendung kommen. So wird für den geriatrischen Patienten definiert, dass eine palliative Kathetereinlage bei einer Harninkontinenz bei „Versagen, Nichtanwendbarkeit oder Ablehnung aller anderen Verfahren“ und bei einer Blasenentleerungsstörung „in Abhängigkeit von dem Restharnvolumen, der Symptomatik und der Koinzidenz mit Infekten oder Hämaturien“ indiziert sein kann [1, 2]. Obwohl auch bei im Eingangsscreening (ISAR-Screening) als „geriatrisch“ markierten Patienten in der Urologie mit Blasenentleerungsstörung [3] unter Einbeziehung des ambulanten Sektors eine suprapubische Kathetereinlage häufig vermieden werden kann [4], verbleibt ein Teil von Patienten, bei denen die Einlage eines Katheters in lebenslanger Intention die einzige sinnvolle Option zur Versorgung einer schweren Harninkontinenz, einer subvesikalen Obstruktion oder z. B. bei einem Mobilitätsverlust darstellt.

Jenseits der Diskussion, welche Form der Katheterableitung mit welchen Kontraindikationen wie Blutverdünnung, Adipositas per magna, Voroperationen im Unterbauch indiziert sein könnte und welche Bedeutung das Geschlecht des Patienten spielt, stellt sich die Frage, auf welcher Datenbasis eine entsprechende Aufklärung des Betroffenen oder seiner Angehörigen im Hinblick auf die Auswirkungen des Katheters auf das weitere Leben beruht. Wenn es etwa gilt, die Risiken einer operativen Desobstruktion oder einer suburethralen Schlingenoperation bei Belastungsinkontinenz bei einem Hochbetagten oder Multimorbiden gegenüber dem Risiko und den langfristigen Folgen der Katheterableitung abzuwägen, sollten naturgemäß nicht nur die Risiken der Ersteinlage eines Katheters mit „Verletzung von Nachbarorganen“ oder „Hämaturien“ und „Infekten“ besprochen werden, sondern auch die Konsequenzen für das weitere soziale Leben des Patienten und seine Lebensqualität auf lange Sicht.

Die Mortalität der Ersteinlage eines SPK wird von Ahluwalia et al. mit 1,8 % [5] und die der Darmverletzung als schwerster und revisionspflichtiger Komplikation von Jacob et al. mit 2,4 % angegeben [6], das Risiko einer komplikativen transurethralen Kathetereinlage mit 6,7 pro 1000 Katheterismen [7]. Allein vor diesem Hintergrund eines relevanten Risikos ist es erstaunlich, dass zu den psychosozialen Folgen einer lebenslangen Kathetereinlage bisher keine Daten vorliegen. Die Untersuchungen, die die Lebensqualität einer Katheterableitung bisher zum Gegenstand hatten, betreffen eine kurzfristige Katheterableitung nach gynäkologischen Operationen [8, 9], nach einer radikalen Prostatektomie [10] oder behandeln ein selektioniertes Patientengut wie bei einer neurogenen Blasenfunktionsstörung [11] z. B. bei Tetraplegie [12]. Die einzige, sich mit ambulant versorgten Katheterträgern beschäftigende Untersuchung basiert auf der Befragung von 27 Katheterträgern (14 weiblich, 13 männlich) mit 10 transurethralen und 17 suprapubischen Kathetern und kann nicht als systematische Untersuchung gewertet werden [13]. Der Aspekt der Lebensqualität wird in einem Review, in dem 14 Untersuchungen unterschiedlicher Patientengruppen und Katheterliegezeiten eingeschlossen wurden, lediglich gestreift [14]. Dagegen existiert zur Untersuchung der Lebensqualität bei Katheterträgern ein validiertes Assessment mit 25 Items, das in der schon genannten Untersuchung im postoperativ-gynäkologischen Setting [9] Anwendung fand [15]. Es ist bisher jedoch noch nie bei einer größeren Patientenzahl und auch noch nie im Hinblick auf die lebenslange Katheteranlage angewendet worden.

Diese Lücke sollte mit der vorliegenden Untersuchung geschlossen werden. Sie stellt die weltweit erste Untersuchung dar, die die Lebensqualität bei Katheterträgern als lebenslange Dauerversorgung zum Gegenstand hat. Durch die Anwendung eines strukturierten, validen Assessments [15] an einer großen Patientenzahl sollte es nicht nur möglich sein, die Lebensqualität unter einer Harnblasenlangzeitdrainage insgesamt in bestimmten Sektoren des täglichen Lebens, sondern auch eventuelle Unterschiede zwischen der transurethralen und suprapubischen Langzeitdrainage, der Indikation zur Anlage des Katheters, dem Alter und dem Geschlecht des Trägers sowie der Katheterdicke herauszuarbeiten.

## Methodik

Verwendung fand das von Wilde et al. inaugurierte Assessment zur Messung der Lebensqualität von Katheterträgern [15]. Es handelt sich um die Adaptation eines Assessments zur Untersuchung der Lebensqualität bei Harninkontinenz [16]. Dieses wurde nach der Übersetzung ins Deutsche Patienten nach Erläuterung und Unterzeichnung einer Datenschutz- und Einverständniserklärung vorgelegt, die bereits mindestens 3 Monate lang einen transurethralen oder suprapubischen Katheter (DK bzw. SPK) in lebenslanger Intention trugen. In der Regel geschah dies durch die Autoren der vorliegenden Arbeit im Rahmen eines Katheterwechsels in der Praxis oder der Klinikambulanz. Zusätzlich zum eigentlichen Fragebogen wurden Daten zur Demographie, Art und Größe des Katheters, seiner Liegedauer und der entsprechenden Indikation erhoben. Der Fragebogen selbst enthält insgesamt 25 Items in 5 Domänen. Diese betreffen Kathetermanagementprobleme (Domäne 09, Angst vor Nässe, Geruch, Infektionen, vor Herausrutschen des Katheters und Probleme, eine Toilette zu finden), interpersonelle Probleme (Domäne 10, Konflikte in Bezug zum Pflegepersonal, zu Ärzten, Angst vor Schmerzen, Probleme in Bezug auf Sexualität und Kleidung), psychosoziale Probleme (Domäne 11, Gefühl von Krankheit, Hilflosigkeit, eingesperrt sein), katheterbezogene technische Probleme (Domäne 12, Angst vor Urinverlust, Lecks, Schmerzen bei Wechsel) und Haut- sowie Schleimhautproblemen (Domäne 13, Sorge vor Haut- bzw. Schleimhautproblemen im Intimbereich). Wegen der Relevanz für geriatrische Patienten wurde der Originalfragenkatalog nach Wilde durch 2 Fragen zu stattgehabten Stürzen ergänzt.

Eingeschlossene Patienten wurden gebeten, die entsprechenden Aussagen wie z. B. „ich bin besorgt, wegen einer Katheterundichtigkeit nass zu werden“ abgestuft mit 5 Punkten zu bewerten: 1 = extreme Zustimmung, 2 = leichte Zustimmung, 3 = moderate Zustimmung, 4 = wenig Zustimmung, 5 = überhaupt keine Zustimmung. Alle Items sind so formuliert, dass hohe Werte eine unbeeinträchtigte Lebensqualität bzw. eine fehlende Besorgnis in diesem Punkt anzeigen, niedrige Werte analog eine stark beeinträchtigte Lebensqualität bzw. starke Sorgen darstellen.

Ein positives Ethikvotum der Ärztekammer Westfalen-Lippe (AZ 2018-075-f-S) lag vor. Ausschlusskriterien waren kognitive Veränderungen, die es nicht möglich machten, auch mit Unterstützung (z. B. durch Verlesen) des Assessments valide Angaben zu erhalten. Die statistische Auswertung wurde durch das Institut Chrestos mit Sitz in Essen vorgenommen. Dabei wurden metrische, annähernd normalverteilte Variablen mit Hilfe des Mittelwerts (MW) und der Standardabweichung (SD) beschrieben. Schiefverteilte Variablen wurden mit dem robusteren Median und dem Interquartilsabstand (IQR) beschrieben. Die Beschreibung kategorialer Variablen erfolgte mit Hilfe absoluter (*n*) und relativer (%) Häufigkeiten.

Die Untersuchung wurde durch eine Förderung der Paul-Kuth-Stiftung der Deutschen Bank durch die Übernahme der Kosten für Datenmanagement und die Statistische Auswertung unterstützt. Eine Einflussnahme auf den Studienentwurf, die statistische Auswertung oder das Publikationsmanuskript bestand nicht.

## Ergebnisse

Insgesamt wurden zwischen Mai 2018 und September 2020 357 Patienten (260 männlich, 97 weiblich) durch Mitglieder des Arbeitskreises „geriatrische Urologie der Dt. Gesellschaft für Urologie“ rekrutiert. Es handelte sich bei 2 fehlenden Angaben um 193 Träger eines SPK (135 männlich, 58 weiblich), 162 Träger eines DK (123 männlich, 39 weiblich).

Das Alter aller Patienten betrug im Mittel 76,5 ± 12,2 Jahre, das der Träger eines SPK 74,4 ± 12,6 Jahre, eines DK 78,9 ± 11,1 Jahre. Dieser Unterschied war im Kruskal-Wallis-Test mit einem *p* < 0,001 statistisch signifikant.

Die Dauer der Katheterableitung betrug zum Zeitpunkt der Datenerfassung median 24 Monate (MW 36,46 ± 42,96 Monate). Die längste Katheterliegedauer betrug 300 Monate oder 25 Jahre.

Die Indikation für die Anlage der Harnableitung in lebenslanger Intention war bei den Trägern eines SPK in 13,6 % eine Harninkontinenz, in 51,6 % eine Blasenentleerungsstörung, bei 33,2 % waren es andere Gründe wie Mobilitätsdefizite, Demenz oder Kontrakturen, bei weiteren 1,6 % war die Indikation unbekannt. Analog ergaben sich für Träger eines DKs als Grund für seine Anlage 19,4 %, 53,8 %, 21,9 % und 5 %. Damit sind die Indikation für die Katheterableitung und die Art des Katheters voneinander abhängig (χ^2^-Unabhängigkeitstest: *p* = 0,032).

Die gewählten Kathetergrößen betrugen bei liegendem SPK in 39,2 % ≤ 14 Ch, 28 % 16 Ch und 32,8 % ≥ 18 Ch, analog ergaben sich für die einliegenden DK 8,4 %, 47,1 % und 44,5 % (Tab. 1). Während damit für den SPK die Kathetergrößen in den gewählten Kategorien nahezu gleich verteilt waren, wurden für die transurethralen Katheter überwiegend 16 Ch oder 18 und mehr Ch eingesetzt. Es zeigte sich mit einem *p* < 0,001 ein signifikanter Einfluss der Kathetergröße nach dem Typ des verwendeten Katheters.

**Table 1:** 

Variable	*n*	SPK	*n*	DK
*Geschlecht (n, %)*	193	–	162	–
Mann	–	135 (69,9)	–	123 (75,9)
Frau	–	58 (30,1)	–	39 (24,1)
*Alter (n, MW, SD)*	191	74,4 ± 12,6	–	78,9 ± 11,1
*Indikation (n, %)*	184	–	–	–
Inkontinenz	–	25 (13,6)	–	31 (19,1)
Blasenentleerungsstörung	–	95 (51,6)	–	86 (53,8)
Andere	–	61 (33,2)	–	35 (21,9)
Unbekannt	–	3 (1,6)	–	8 (5,0)
*Größe (n, %)*				
≤ 14 Ch	–	73 (39,2)	–	13 (8,4)
16 Ch	–	52 (28,0)	–	73 (47,1)
≥ 18 Ch	–	61 (32,8)	–	69 (44,5)

*DK* Dauerkatheter, *SPK* suprapubischer Harnblasenkatheter, *MW* Mittelwert, *SD* Standardabweichung

### Beurteilung der Lebensqualität auf Domänenebene

Insgesamt ließ sich für alle Fragen der 5 Domänen (außer dem hinzugefügten Fragenkomplex bzgl. eventueller Stürze) ein kumulativer Lebensqualitätsscore von median 4,4 und im Mittel von 4,1 ± 0,9 ermitteln. Damit liegt der mittlere Lebensqualitätsscore in der vorliegenden Untersuchung höher als in der genannten Arbeit von Wilde mit 3,27 Punkten bzw. auf der zugrunde liegenden Skala von 1 (maximal eingeschränkte Lebensqualität) und 5 (überhaupt nicht eingeschränkte Lebensqualität) bzw. eine nur moderat eingeschränkte Lebensqualität.

**Figure 1:**
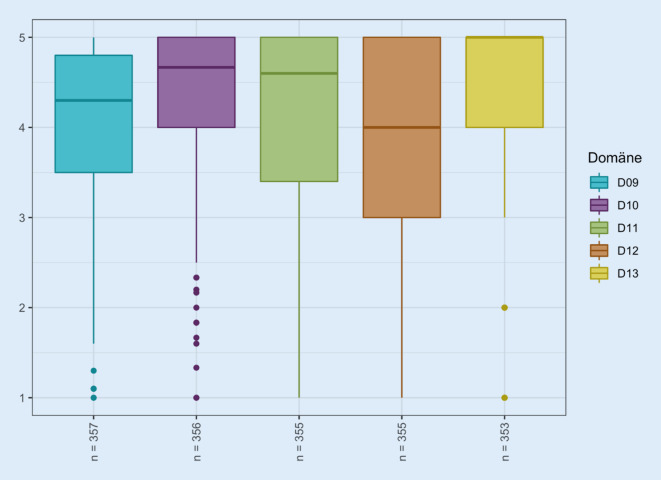

Bezogen auf die Einzeldomänen zeigt sich für die Kathetermanagementdomäne (Domäne 09) ein kumulativer Lebensqualitätsscore von median 4,3 (im Mittel 4,0 ± 0,9), für die Domäne der interpersonellen Probleme (Domäne 10) von 4,7 (im Mittel 4,4 ± 0,8), für die psychosozialen Probleme (Domäne 11) von 4,6 (MW 4,1 ± 1,2), für die katheterbezogene Lebensqualität (Domäne 12) von 4,0 (MW 3,8 ± 1,2) und die Haut- bzw. Schleimhautprobleme (Domäne 13) von 5,0 (MW 4,2 ± 1,3). Im Median liegen die stärksten Einschränkungen der Lebensqualität in den Domänen der Kathetermanagementprobleme (D 09) und der katheterbezogenen Lebensqualität (D 12; Abb. 1).

Dabei zeigt die Betrachtung der Ergebnisse der Einzelfragen im Vergleich zu dem Gesamtscore der einzelnen Domäne, dass – mit unterdurchschnittlichem Punktwert im Vergleich zum Domänenscore – v. a. Sorgen im Kontext einer möglichen Urinleckage (Frage 9a, 3,83 ± 1,49) mit Angst davor, dass andere Uringeruch wahrnehmen könnten (9e, 3,78 ± 1,4), bestehen. Es wird eine Angst vor Harnwegsinfektionen (9h, 3,50 ± 1,57) und Sorge um die damit verbundene erforderliche Trinkmenge (9g, 3,96 ± 1,53) angegeben. Katheterträger haben zusätzlich vermehrt Sorgen, dass sich die Probleme im Alter vergrößern könnten (9f, 3,90 ± 1,43) und äußern, ihr Leben wegen des Katheters weniger genießen zu können (11b, 3,92 ± 1,42). Sie sind frustriert, dass sie der Katheter davon abhält, zu tun, was sie mögen (11c, 3,99 ± 1,42). Es besteht das Gefühl, dass die Auswahl ihrer Kleidung begrenzt ist (10f, 4,08 ± 1,37). Katheterträger äußern vermehrt Angst vor möglicherweise schmerzhaften Katheterwechseln (12c, 3,83 ± 1,28) und geben auch an, dass sie sich durch den Katheter als kranke Person fühlen (11a, 4,01 ± 1,39). Die Angst vor Uringeruch und vor einer Harnwegsinfektion zeigen dabei mit 3,78 bzw. 3,50 im Mittel die niedrigsten Punktwerte an.

### Geschlechterunterschiede

Die Unterschiede in der Beurteilung der einzelnen Fragen gruppiert nach Geschlecht zeigt einen Median von 4,4 für Männer (MW 4,2 ± 0,8) und von 4,3 für Frauen (MW 4,0 ± 0,9). Damit finden sich nur geringe Unterschiede in der Beurteilung der Lebensqualität bei weiblichen und männlichen Katheterträgern. Diese wird von Frauen tendenziell schlechter beurteilt.

Es ergibt sich eine Geschlechterabhängigkeit in den Fragen 9b (Suche nach geeigneter Toilette, *p* = 0,001), e (Sorge vor Uringeruch, *p* = 0,036) und f (Sorge vor größeren Katheterproblemen im Alter, *p* = 0,017). Ebenso findet sich eine Geschlechterabhängigkeit bei dem Gefühl, das Zuhause wegen des Katheters nicht mehr für längere Zeit verlassen zu können (11e, *p* = 0,013), bei unfreiwilligem Urinverlust (Frage 12b, *p* = 0,012) und bei Hautproblemen im Unterbauch/Intimbereich (Frage 13, *p* = 0,008). Es zeigt sich bei Betrachtung der relativen Häufigkeiten der Beantwortung der Fragen tendenziell eine schlechtere Bewertung bei Frauen in den genannten Bereichen.

### Kathetertyp

**Figure 2:**
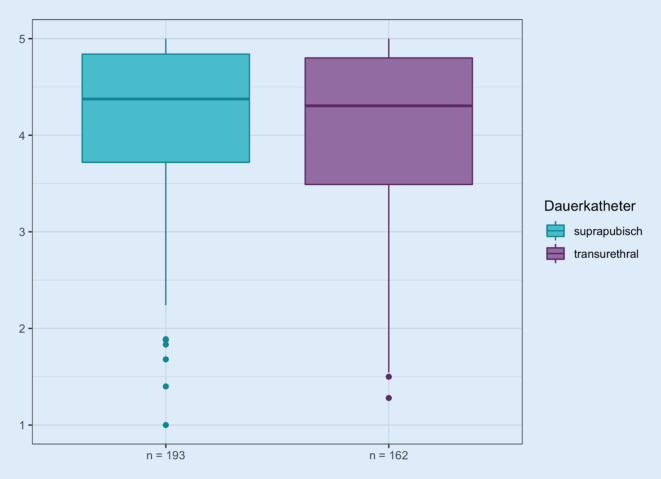

Die Gesamtauswertung aller Fragen in den Domänen 9–13 zeigt gruppiert für die transurethrale und suprapubische Harnableitung aller Patienten einen medianen Score von 4,4 bzw. 4,3 bei einem MW von 4,2 ± 0,8 bzw. 4,0 ± 0,9 (Abb. 2). Damit ist im Mittel der Gesamtlebensqualitätsscore bei Patienten mit DK sehr gering schlechter als im Vergleich zu SPK-Patienten.

Die Tabelle A2 im Anhang zeigt die Ergebnisse der Befragung in den einzelnen Domänen bzw. Fragen kategorisiert nach der Art der Harnableitung per suprapubischem oder transurethralem Katheter. Dargestellt wird die kategorisierte Betrachtung der Antworten (1 = extreme Zustimmung, 2 = leichte Zustimmung, 3 = moderate Zustimmung, 4 = wenig Zustimmung, 5 = überhaupt keine Zustimmung) in Prozent mit dem *p*-Wert des χ^2^-Unabhängigkeitstests. Signifikante Werte im χ^2^-Unabhängigkeitstest deuten damit auf eine Abhängigkeit der Antworten der entsprechenden Frage von der Art der Katheterableitung hin.

Damit findet sich bezogen auf die Gesamtheit aller Patienten keine signifikante Abhängigkeit in den 25 Fragen der 5 Domänen bezüglich eines Unterschieds zwischen der Art der Katheterableitung.

### Kathetertyp und Geschlecht

**Figure 3:**
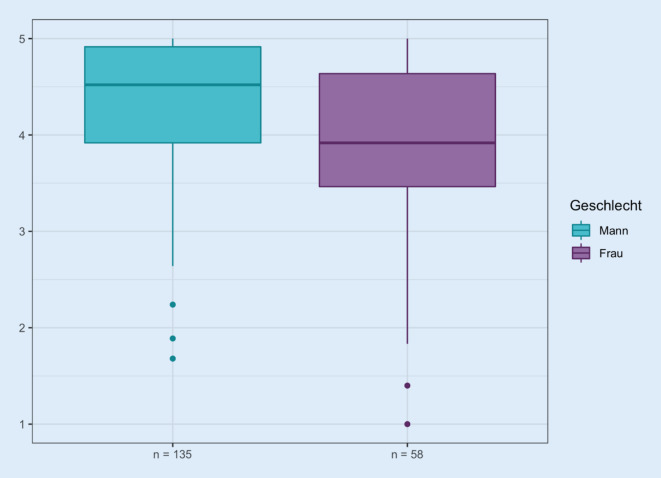

Stratifiziert nach Geschlecht zeigt sich für weibliche Träger eines suprapubischen Katheters eine mediane Beurteilung der Lebensqualität von 3,9 (MW 3,9 ± 1,0) Punkten, für Männer von 4,5 (MW 4,3 ± 0,7) Punkten. Dahingegen fällt die Beurteilung der Lebensqualität, wenn ein transurethraler Katheter getragen wird, mit einem Median von 4,3 Punkten für Männer und einem Median von 4,3 Punkten für Frauen (MW für Männer 4,0 ± 0,9; für Frauen 4,1 ± 0,8) ähnlich aus (Abb. 3). Offenbar bewerten damit im Mittel Trägerinnen eines SPK ihre globale Lebensqualität schlechter als Männer mit einem SPK, während sich solche Unterschiede bei Trägern eines DK nicht nachweisen lassen.

**Figure 4:**
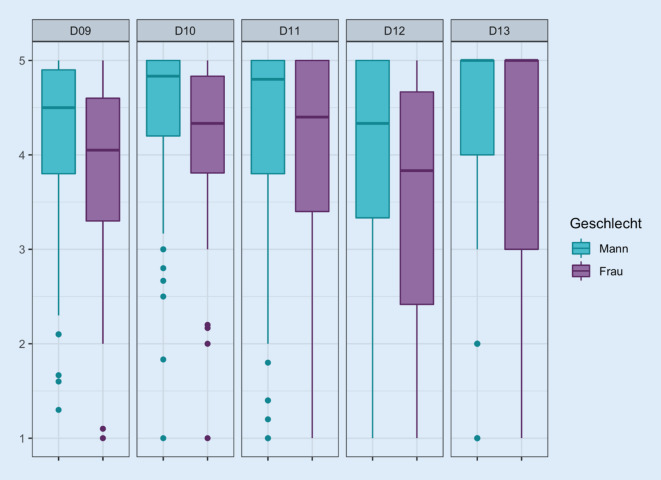

**Figure 5:**
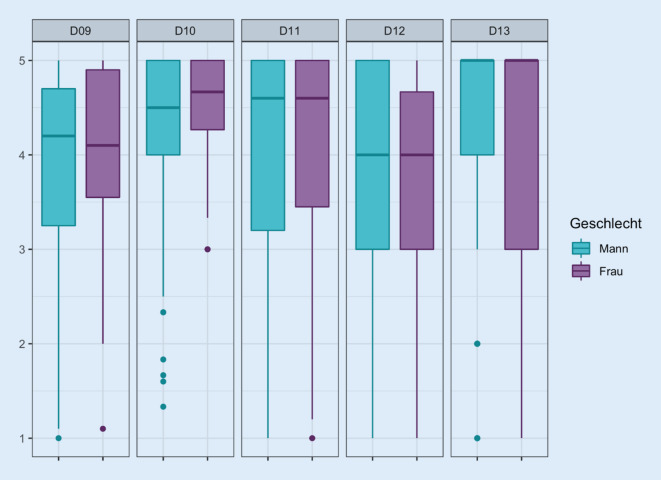

Frauen mit einem suprapubischen Katheter geben tendenziell für alle Domänen mit Ausnahme der Domäne 13 („Haut/Schleimhautprobleme“) niedrigere Punktwerte, d. h. eine schlechtere Beurteilung der domänenbezogenen Lebensqualität als Männer mit SPK an (Abb. 4). Dahingegen bestehen nur marginale Unterschiede in der Angabe der Lebensqualität auf Domänenebene bei männlichen bzw. weiblichen Trägern eines transurethralen Katheters (Abb. 5).

Die statistische Analyse zeigt eine Abhängigkeit der Antworten der Fragen 9b, 9e, 9i, 10f und 11e vom Geschlecht. Beim Vergleich der relativen Häufigkeiten der Antworten in den Gruppen Männer und Frauen werden bei den Frauen bei den Fragen 9b („ich bin besorgt, in der Nähe eine Toilette zu finden“, *p* = 0,003), bei der Frage 9e (Sorge vor Uringeruch, *p* = 0,024), bei der Frage 9f (größere Katheterprobleme im Alter, *p* = 0,021) und 9i (Sorge, den Katheterbeutel rechtzeitig leeren zu können, *p* = 0,013), bei der Frage 10f (eingeschränkte Auswahl der Kleidung, *p* = 0,007), 11e (Gefühl, das Zuhause nicht für längere Zeit verlassen zu können, *p* = 0,003), 12b (Sorge vor unfreiwilligem Urinverlust) und 13 (Angabe von Haut/Schleimhautproblemen, *p* = 0,006) häufiger eine Zustimmung gegeben als bei den Männern und damit eine Einschränkung der entsprechenden Lebensqualität signalisiert. Im Gegensatz dazu zeigt die Statistik eine Abhängigkeit vom Geschlecht bei Trägern eines transurethralen Dauerkatheters lediglich bei den Fragen 9d (Katheterblock, *p* = 0,028) und 10f (Kleiderauswahl, *p* = 0,016).

### Grund der Katheterableitung

**Figure 6:**
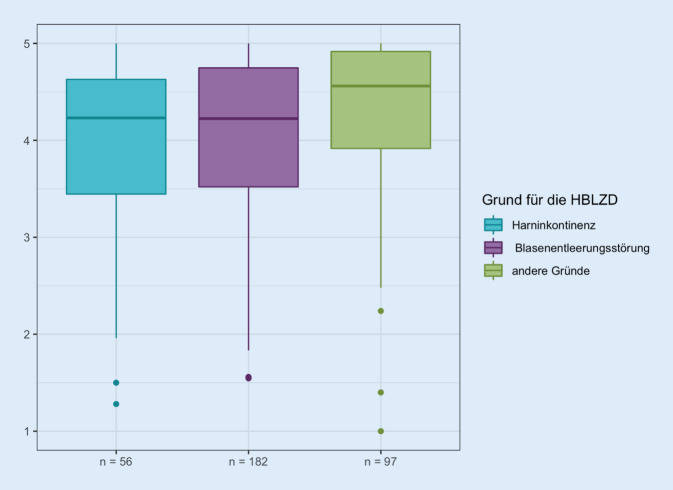

Die Gesamtauswertung der Lebensqualitätsscores in Abhängigkeit von der Indikation demonstriert für Patienten mit einer Harninkontinenz als Indikation einen Median von 4,2 (MW 4,0 ± 0,9), für die Patienten mit einer zugrunde liegenden Blasenentleerungsstörung von 4,2 (MW 4,0 ± 0,9) und für solche mit anderen Indikationen wie z. B. Immobilität oder Multimorbidität einen Median von 4,6 (MW 4,2 ± 0,8; Abb. 6). Im Mittel zeigen Patienten mit Indikationsgrund Harninkontinenz und Blasenentleerungsstörung die gleiche Lebensqualität. Patienten mit anderen Indikationsgründen haben hingegen im Vergleich im Mittel eine leicht bessere Lebensqualität.

**Figure 7:**
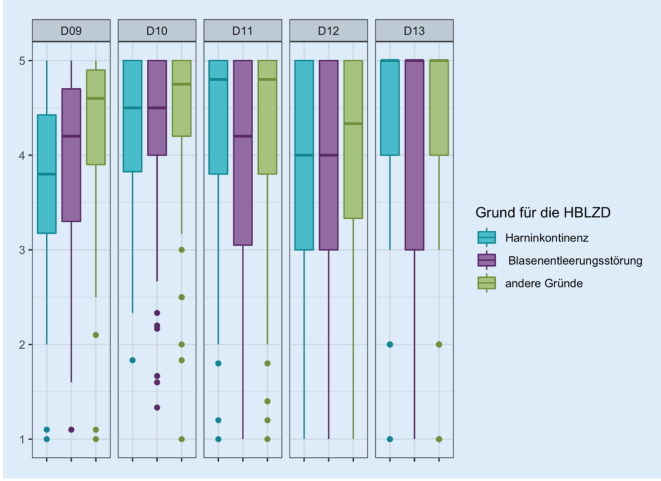

Auf der Domänenebene finden sich in den Domänen 9 (Kathetermanagementbewertung), 10 (interpersonelle Probleme) und 12 (katheterbezogene Lebensqualität) höhere mediane Lebensqualitätsscores für die Indikation der Katheteranlage mit „anderen Gründen“ im Vergleich zu Harninkontinenz und Blasenentleerungsstörung (Abb. 7).

Es zeigt sich eine Abhängigkeit zwischen den Beantwortungen der Fragen 9e (*p* < 0,025), 9f (*p* = 0,025), 9g (*p* < 0,001), 9h (*p* < 0,047), 9i (*p* = 0,003), 10a (*p* = 0,048), 10f (*p* = 0,025) und 11c (*p* = 0,036) und Indikation für den Katheter.

Es zeigt sich, dass der relative Anteil an Patienten, die eine extreme Zustimmung zu den Items „Angst vor Uringeruch“ (Frage 9e) „Angst vor Harnwegsinfektionen“ (9h) und „Sorge, die Beutelentleerung rechtzeitig vornehmen zu können“ (9i) in der Gruppe der Patienten mit Harninkontinenz höher ist als in den Gruppen der Patienten mit Blasenentleerungsstörung oder einer Indikation aus anderen Gründen. Ebenso findet sich ein höherer Anteil an Patienten in den genannten Gruppen, die eine extreme Zustimmung zu der betreffenden Frage formulieren in den Items „Probleme mit zunehmendem Alter“ (9f), „Sorge um ausreichende Trinkmenge“ (9g) und „Kommunikation mit Pflegepersonal“ (10a). Dahingegen ist der relative Anteil von Patienten, die eine extreme Zustimmung in den Punkten „Auswahl der Kleidung“ und in ihrer „Freizeitgestaltung“ in der Gruppe der Patienten mit Blasenentleerungsstörung höher (10f und 11c).

### Kathetergröße

Es fand sich in der statistischen Analyse eine nicht zufällige Verteilung der einliegenden Kathetergröße von dem Geschlecht der Katheterträger, der Katheterart und seiner Indikation. Es tragen mehr Männer Kathergrößen von ≤ 14 Ch und 16 Ch, bei Frauen werden häufiger Katheter von ≥ 18 Ch verwendet. 84,9 % der Patienten mit einer Kathetergröße von ≤ 14 Ch haben einen SPK, aber nur 15,1 % haben einen DK. Der Anteil von Patienten mit einem SPK ist hingegen in der Gruppe der Kathetergröße 16 bzw. ≥ 18 mit 41,6 % bzw. 46,9 % geringer als der Anteil der Patienten mit einem DK (58,4 % bzw. 53,1 %) in diesen Gruppen. Kathetergrößen von ≥ 18 Ch finden am häufigsten bei einer Harninkontinenz und anderen Indikationen Verwendung, bei einer Blasenentleerungsstörung sind es Kathetergrößen ≤ 14 Ch (Tab. 2).

**Table 2:** 

Variable	≤ 14 Ch	16 Ch	≥ 18 Ch	*p*
*Geschlecht (n [%])*				
Mann	59 (67,8)	102 (81,6)	92 (17,2)	0,041
Frau	28 (32,2)	23 (18,4)	39 (29,8)	n. s.
*Alter (Jahre [MW, SD])*	73 ± 13,8	77,2 ± 11,9	77,4 ± 11,1	n. s.
*Katheterart (n [%])*				
Suprapubisch	73 (84,9)	52 (41,6)	61 (46,9)	< 0,001
Transurethral	13 (15,1)	74 (58,4)	59 (53,1)	n. s.
*Indikation (n [%])*				
Harninkontinenz	8 (9,6 %)	19 (15,3 %)	25 (19,7 %)	< 0,001
Blasenentleerungsstörung	60 (72,3 %)	64 (51,6 %)	53 (41,7 %)	n. s.
Andere	12 (14,5 %)	36 (29,0 %)	48 (37,8 %)	n. s.
Unbekannt	3 (3,6 %)	5 (4,0 %)	1 (0,8 %)	n. s.

*MW* Mittelwert, *SD* Standardabweichung, *n. s.* nicht signifikant

Der Gesamtlebensqualitätsscore bei Patienten mit einem Katheter ≤ 14 Ch beträgt median 4,3 (MW 4,1 ± 0,8), bei Patienten mit einem Katheter der Größe 16 Ch 4,4 (MW 4,2 ± 0,8) und bei solchen mit einem Katheter ≥ 18 Ch median 4,5 (MW 4,1 ± 1,0) und ist damit vergleichbar.

Bei insgesamt 3 Fragen zeigte sich eine nicht zufällige Verteilung der Antworten von der Kathetergröße (Fragen 9h, *p* = 0,002, 12b, *p* = 0,036, und 11c, *p* = 0,012). Es zeigte sich bei der Betrachtung der relativen Häufigkeiten der Antworten innerhalb der Gruppen, dass eine extreme Zustimmung der Fragen 9h (Sorge um Harnwegsinfektionen) und 12b (unfreiwilliger Urinverlust) bei einer Kathetergröße von ≥ 18 Ch häufiger getroffen wird als im Vergleich zu den andern Kathetergrößen.

Die Aussage 11c (Frustration über Einschränkungen der Freiheit zu tun, was der Betroffene will) hingegen wird von Trägern mit einem Katheter ≤ 14 Ch häufiger mit einer extremen Zustimmung belegt als im Vergleich mit anderen Kathetergrößen.

### Alter

**Figure 8:**
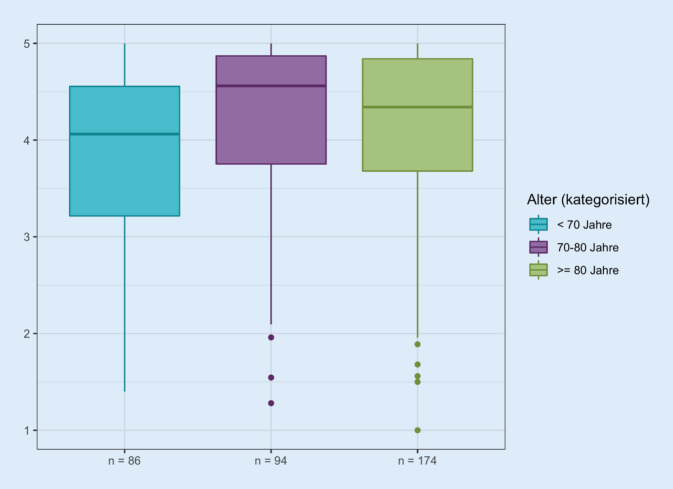

Es ergibt sich ein Gesamtpunktwert der gemessenen Lebensqualität von median 4,1 (MW 3,9 ± 0,9) Punkten für die Patienten unter 70 Jahren, von 4,6 (MW 4,2 ± 0,8) in der Altersgruppe 70 bis 80 Jahre und in der Altersgruppe ≥ 80 Jahre von 4,3 (MW 4,2 ± 0,8; Abb. 5). Damit zeigt sich eine Tendenz zu einer schlechter bewerteten Lebensqualität bei den Patienten < 70 Jahre (Abb. 8).

Es zeigt sich bei Betrachtung der relativen Häufigkeiten der Beantwortung der Fragen tendenziell eine schlechtere Bewertung bei Patienten < 70 Jahre im Vergleich zu den anderen Altersgruppen in den Bereichen „Sorge vor Uringeruch“ (Frage 9e, *p* = 0,003), „Sorge, dass die Probleme im Alter zunehmen könnten“ (9f, *p* = 0,006), „Beeinträchtigung der Sexualität“ (10e *p* = 0,001) und „Auswahl der Kleidung“ (10f, *p* = 0,036), „Sorge, das Leben wegen des Katheters weniger genießen zu können“ (11b, *p* = 0,011) und „Frustration darüber, dass der Katheter davon abhält, zu tun, was man mag“ (11c, *p* = 0,006).

### Stürze

Es gaben 14,8 % der Befragten (*n* = 352, n.a. = 5) an, im letzten Jahr vor der Befragung mehrfach gestürzt zu sein, wegen des Katheters jedoch nur 0,6 % (*n* = 351, n.a. = 6). Die Häufigkeit dieser Angabe ist für beide Fragen unabhängig von der Art der Harnableitung i. S. eines transurethralen oder suprapubischen Katheters (*p* = 0,880 bzw. 0,503), dem Grund für die Harnableitung (Harninkontinenz, Blasenentleerungsstörung, andere, *p* = 0,779 bzw. 0,714) und das Alter (*p* = 0,437 bzw. 0,362).

## Diskussion

Wilde et al. entwickelten und validierten 2010 [15] in Rochester, NY, ein Assessmentinstrument zur Evaluation der katheterabhängigen Lebensqualität bei Trägern eines Katheters in lebenslanger Intention. In diesem Assessment sind 25 Fragen in 5 Domänen enthalten, die skaliert mit „extremer Zustimmung“ bis zu „überhaupt keine Zustimmung“ einen Punktwert von zwischen 1 (maximale Beeinträchtigung) und 5 (keine Beeinträchtigung) ergeben. Wilde et al. untersuchten in einer Pilotstudie 11 Patienten mit transurethralem Katheter und in einer weiteren kleinen prospektiven Untersuchung 43 Katheterträger zu Beginn der Katheterableitung, nach 2, 4 und nach 6 Monaten im Hinblick auf die Reliabilität. Es ergaben sich jeweils MW zwischen 3 und 3,7 Punkten, die auf eine geringe bis moderate Einschränkung der Lebensqualität durch den Katheter hindeuten.

Dieses katheterspezifische Assessment wurde nach Kenntnis der Autoren seitdem nicht erneut eingesetzt – die Fragestellung der Lebensqualität von Personen mit lebenslanger Katheterableitung wurde seitdem auch nicht mehr untersucht. Dies ist umso erstaunlicher, da doch die Katheteranlage als Palliativmaßnahme z. B. bei einer Harninkontinenz, einer Blasenentleerungsstörung oder Mobilitätsverlusten eine gängige und vielgeübte Praxis darstellt, ohne dass hierzu genaue Versorgungsforschungsdaten existieren. Während die Einlage eines transurethralen Katheters häufig als vorübergehende Maßnahme z. B. im Rahmen einer Operation, einer schweren Allgemeinerkrankung oder im Krankenhaus zur Bilanzierung initiiert wird und dann häufig im weiteren Verlauf durch fehlende Rekonvaleszenz oder Mobilisierung in eine lebenslange Katheterableitung übergeht, stellt die Anlage eines suprapubischen Katheters entweder als Wechsel der Ableitung bei schon länger liegendem transurethralen Dauerkatheter oder prima vista einen operativen Eingriff dar, der einer Lokalanästhesie oder einer Allgemeinnarkose bedarf. Dieser Eingriff beinhaltet die Aufklärung mit forensisch wichtigen Aspekten wie die Komplikationen einer Blutung oder Verletzung von Nachbarorganen. Die in der Literatur angegebenen Daten einer Mortalität von 1–2 % lassen den Eingriff als nicht banal erscheinen [5, 6].

Neben diesen technischen Aspekten bleibt die Frage, auf welcher Datenbasis die Aufklärung jenseits der Risiken der Ersteinlage und der bekannten Langzeitfolgen einer Langzeitdrainage wie Hämaturien, Infekte oder Steinbildungen im Hinblick auf die Folgen für das weitere Leben, die Freizeitgestaltung, die Kleidung und Sexualität und anderen Aspekten überhaupt beruht. Diese Einschränkungen wären gegen die Vor- und Nachteile sowie die Risiken von Alternativen abzuwägen. So ist zumindest bei der Harninkontinenz eine Versorgung mit aufsaugenden Hilfsmitteln oder einem Kondomurinal bei Männern denkbar – bei einer Blasenentleerungsstörung etwa durch ein benignes Prostatasyndrom wären diese Aspekte dem Risiko einer desobstruierenden Operation mit ihren urologischen und allgemeinen Konsequenzen gegenüberzustellen. Dies erscheint umso wichtiger, da eine Katheteranlage als Dauerversorgung nach Monaten der Dauerableitung mit hoher Wahrscheinlichkeit durch funktionelle und morphologisch-anatomische Folgen nicht mehr reversibel ist.

Die vorliegende Untersuchung beschäftigt sich erstmals mit der katheterbezogenen Lebensqualität bei Katheterträgern als lebenslange Dauerversorgung unter Verwendung des genannten, bereits existierenden Assessments von Mary Wilde. Einschlusskriterien waren die als lebenslange Dauerversorgung intendierte Harnblasenlangzeitdrainage, die seit mindestens 3 Monaten etabliert sein sollte, auch um „Katheterwechselpatienten“ auszuschließen. Der Erhebungsbogen wurde professionell ins Deutsche übersetzt – auf eine Validierungsstudie, für die keine Mittel zur Verfügung standen, wurde verzichtet.

Es können auf dem Boden der vorliegenden Ergebnisse folgende Aussagen getroffen werden:

- Die vorliegende Untersuchung zeigt auf einer Skala von 1–5 Punkten insgesamt mit einem Median von 4,4 und einem MW von 4,1 ± 0,9 Punkten eine nur mäßige Einschränkung der *katheterbezogenen Lebensqualität*.
- Dieser Wert liegt höher als in der Originalpublikation von Wilde mit im Mittel 3,27. Dies könnte neben regionalen Unterschieden vor allem der geringen Gruppengröße von nur 43 Probanden bei Wilde gegenüber 357 in der vorliegenden Untersuchung geschuldet sein.
- Die größten *Einschränkungen der Lebensqualität* zeigen sich in der vorliegenden Untersuchung in den Domänen 9 (Kathetermanagementprobleme) und 12 (katheterbezogene Lebensqualität).
- Katheterträger weisen mit einer schlechteren Bewertung ihrer Lebensqualität in den Einzelitems im Vergleich zu den Domänenscores eine Beeinträchtigung in folgenden Bereichen auf:
  - Sorge vor Urinleckagen,
  - Angst vor Uringeruch,
  - Angst vor Harnwegsinfektionen,
  - Sorge um ausreichende Trinkmenge,
  - Sorge, dass katheterbezogene Probleme im Alter größer werden können,
  - Sorge, dass sie ihr Leben weniger genießen können,
  - Frustration, dass sie der Katheter davon abhält, zu tun, was sie mögen,
  - Auswahl der Kleidung,
  - Angst vor schmerzhaften Katheterwechseln.
- Dabei weisen die Items „Angst vor Uringeruch“ und „Angst vor Harnwegsinfektionen“ die schlechtesten Bewertungen auf.
- Es findet sich eine geringgradig schlechter beurteilte *globale Lebensqualität* bei Frauen als bei Männern. Dieses betrifft v. a. inkontinenzbezogene Probleme aber auch zusätzlich eine größere Sorge vor Problemen mit der Haut im Unterbauch bzw. Intimbereich.
- Der globale *Lebensqualitätsscore bei Frauen* mit einem suprapubischen Katheter liegt im Vergleich mit Männern mit einem SPK niedriger.
- Im Gegensatz dazu wird die Lebensqualität bei mit einem *transurethralen Katheter* versorgten Männern wie Frauen nahezu identisch beurteilt.
- Die Unterschiede in der Beurteilung der Lebensqualität bei Trägern eines SPK sind in nahezu allen Domänen ablesbar; im χ^2^-Unabhängigkeitstest wird eine signifikante Abhängigkeit vom Geschlecht bei den Fragen aufgezeigt, die um die Problematik eines unfreiwilligen Urinverlusts und den damit verbundenen Problemen kreisen. So haben mit einem SPK versorgte Frauen mehr Angst vor Uringeruch, vermehrte Sorgen, eine Toilette rechtzeitig zu finden, fühlen sich in der Auswahl der Kleidung mehr behindert und unterliegen verstärkt dem Gefühl, das Zuhause nicht mehr für längere Zeit verlassen zu können. Dahingegen fällt die Beurteilung der globalen Lebensqualität und die in den einzelnen Domänen zwischen Männern und Frauen mit einem DK sehr ähnlich aus.
- Damit zeigt die vorliegende Untersuchung erstmals für Frauen mit einem suprapubischen Katheter eine schlechtere Beurteilung ihrer Lebensqualität als bei entsprechend versorgten Männern. Dieser Effekt ist am ehesten durch persistierende bzw. neu aufgetretene transurethrale Urinverluste bei liegendem SPK zu erklären, die es bei Männern mit einem SPK aus anatomischen oder funktionellen Gesichtspunkten heraus so offenbar nicht gibt. Da bei Männern als Grund für eine palliative Katheteranlage häufiger eine Blasenentleerungsstörung z. B. bei einem benignen Prostatasyndrom zu vermuten ist als bei Frauen und Männer als Inkontinenztyp selten eine Belastungsinkontinenz aufweisen, sind sie seltener von einem transurethralen Urinverlust bei liegendem SPK betroffen.
- Die Lebensqualität wird von Katheterträgern je nach der Indikation für eine *Katheterableitung* unterschiedlich beurteilt.
- Die *globalen Lebensqualitätsscores* liegen niedriger, wenn die Indikation für die lebenslange Kathetereinlage mit einer Harninkontinenz oder einer Blasenentleerungsstörung im Harntrakt begründet war.
- Bei der Indikation für die Katheterableitung geben Patienten mit einer Harninkontinenz signifikant häufiger Einschränkungen ihrer Lebensqualität in den typischen Bereichen wie Angst vor Geruch, Leckage, Sorge, einen vollen Katheterbeutel rechtzeitig leeren zu können, an. Sie haben im Gegensatz zu Patienten mit anderen Indikationen Bedenken, dass sich die Probleme im Alter verstärken könnten und mehr Angst vor Harnwegsinfektionen. Dies schlägt sich offenbar in einer größeren Sorge, Betreuungspersonen über die richtige Katheterpflege zu informieren, nieder. Da bei einer Harninkontinenz als Indikation für eine Katheteranlage sowohl bei einer Überaktiven Blase als auch einer stärkergradigeren Belastungsinkontinenz mit Sphinkterinsuffizienz und sogar bei einer extraurethralen Inkontinenz Urinabgänge neben einem transurethralen Katheter oder bei liegendem suprapubischem Katheter durch das Fistelstoma oder per urethram möglich sind, bildet das gewählte Assessment hier diese Problematik ab. Dieses wiegt umso schwerer, da es bei einer jahre- oder jahrzehntelangen Katheterableitung durch den chronischen Infekt in Kombination mit der Schleimhautirritation durch das Kathetermaterial selbst zu einer OAB-Symptomatik mit Tenesmen kommen kann.
- Die Beurteilung der *katheterassoziierten Lebensqualität* hängt vom Alter ab.
- Bei den Items „Sorge über Urinlecks“, „Uringeruch“, „Sorge um eine Beeinträchtigung der Sexualität“ und „Probleme bei der Auswahl der Kleidung“ besteht in der statistischen Analyse eine Abhängigkeit vom Alter. Es geben hier besonders jüngere Patienten unter 70 Jahren eine schlechtere Bewertung der Lebensqualität in diesen Bereichen an. Dies wird auch an der öfter getroffenen Zustimmung zu der Aussage, dass der Katheter jüngere Patienten von einer freien Lebensgestaltung abhält und sie das Leben weniger genießen können, deutlich.
- Es lassen sich Einflüsse der *Kathetergröße* auf die Lebensqualität messen.
- Patienten mit einer Kathetergröße von ≥ 18 Ch geben häufiger eine Sorge vor unfreiwilligem Urinverlust an und sind auch stärker besorgt wegen einer Harnwegsinfektion; wohingegen Patienten mit einem kleineren Katheterdurchmesser von 14 Ch oder weniger sich besorgt darüber zeigen, dass der Katheter sie davon abhält, zu tun, was sie wollen.
- Der Grund für die gefundenen Effekte könnte sein, dass es sich um unterschiedliche Stadien der Katheterableitung handelt bzw. die offenbar bestehende Urinverlustproblematik bereits durch (mehrfache) Wechsel zu einem dicklumigeren Katheter versucht wurde, zu begegnen. Hier ist zu vermuten, dass es sich bei Patienten mit großem Katheterdurchmesser um solche handelt, bei denen möglicherweise frustran versucht wurde, ein Fistelstoma „abzudichten“ bzw. den transluminalen Urinabfluss zu optimieren.
- Mit 14,8 % gab jeder 6. Befragte an, im Jahr vor der Befragung gestürzt zu sein, wegen des Katheters jedoch nur 0,6 % oder jeder 166. Patient. Damit ist die Häufigkeit von Stürzen vergleichbar mit Untersuchungen, die das Risiko von Stürzen bei Patienten mit Harninkontinenz untersuchen [17]. Hier steht die bisher nicht in der Literatur belegte Annahme im Raum, dass der Katheter selbst bzw. das Ableitungssystem als „Stolperfalle“ zu Stürzen führen könnte und nicht die zugrunde liegende Multimorbidität, die Sarkopenie oder ein Schwindelsyndrom. Das in der vorliegenden Untersuchung selten getroffene Statement, wegen des Katheters gestürzt zu sein, könnte als Hinweis darauf gewertet werden, dass nicht der Katheter selbst das Sturzrisiko erhöht, sondern als Indikator für Frailty und nicht zuletzt auch Multimedikation gewertet werden muss.
- Insgesamt zeigt die erste Untersuchung der katheterbezogenen Lebensqualität an einer großen Patientengruppe eine mäßige Einschränkung der Gesamtlebensqualität. Diese ist bestimmt von der Sorge vor technischen Katheterproblemen mit Katheterlecks, Uringeruch, Angst vor Infektionen, die dann wiederum zu Einschränkungen in der Lebensgestaltung führen. Betroffen sind insbesondere Frauen, jüngere Patienten und solche mit einer Harninkontinenz als Grund für die lebenslange Kathetereinlage.
- Bei jüngeren Patienten kommt auch die Angst vor Einschränkungen der Sexualität und Auswahl der Kleidung hinzu. Die genannten Aspekte sollten – neben den technischen Aspekten der Einlage eines Katheters selbst – in die Aufklärung eines Patienten einfließen, wenn die lebenslange Katheteranlage als Alternative zu anderen konservativen oder auch operativen Maßnahmen mit dem Patienten und seinen Angehörigen besprochen werden. Besonders bei jüngeren Patienten und Patienten mit Harninkontinenz sollten Behandler die Formulierung der Leitlinie Harninkontinenz bei geriatrischen Patienten mit „… wenn alle anderen Therapieoptionen nicht anwendbar sind, versagt haben oder nicht gewünscht werden“ beherzigen und besonders die letzte dieser Bedingungen erst nach Aufklärung des Patienten in Kenntnis der vorliegenden Untersuchung gelten lassen.

## Fazit für die Praxis

- In lebenslanger Indikation ist die Lebensqualität insgesamt nur moderat durch die Harnableitung per Katheter eingeschränkt – bei weiblichen Katheterträgern stärker als bei männlichen, bei jüngeren Katheterträgern stärker als bei älteren.
- Stärkere Einschränkungen in der katheterbezogenen Lebensqualität werden durch Urinleckagen (dies besonders bei Frauen und, wenn Harninkontinenz die Indikation für die Katheteranlage war), Angst vor Uringeruch und schmerzhaften Katheterwechseln, Beschränkungen in der Kleidungswahl und Freizeitgestaltung angegeben.
- Die Lebensqualität wird von Frauen mit SPK schlechter beurteilt als von Männern mit SPK, bei DK-versorgten Patienten zeigen sich keine Unterschiede.

## Supplementary Information




