# Supplementary material for: Quality of life for wearers of a suprapubic or transurethral bladder catheter as lifelong permanent care
Source: Urologe A. 2021 Oct 4;61(1):18–30. [Article in German] doi: 10.1007/s00120-021-01642-1 (PMC8763733; doi:10.1007/s00120-021-01642-1)
Supplement: Supplementary file 1 [file 120_2021_1642_MOESM1_ESM.docx]

| **Domäne** | **Frage** | **n =** | **Me-dian** | **Mittel-wert** | **SD** | **IQR** |
| --- | --- | --- | --- | --- | --- | --- |
| Katheter-Management-Probleme  (LQDS 4,0) | „Ich bin besorgt, wegen einer Katheterundichtigkeit nass zu werden“ (9a) | 354 | 5 | **3,83** | 1,49 | 2,75 |
|  | „Ich bin besorgt, in der Nähe eine Toilette zu finden“ (9b) | 352 | 5 | 4,38 | 1,15 | 1,00 |
|  | „Ich bin besorgt, öffentliche Toiletten zu finden, die geeignet sind, dass ich dort den Katheterbeutel leeren kann“ (9c) | 347 | 5 | 4,36 | 1,20 | 1,00 |
|  | „Ich bin besorgt über den Katheterblock“ (9d) | 331 | 5 | 4,25 | 1,27 | 1,00 |
|  | „Ich bin besorgt, dass andere den Uringeruch an mir wahrnehmen könnten“ (9e) | 348 | 5 | **3,78** | 1,47 | 2,00 |
|  | „Ich bin besorgt, dass die Katheterprobleme größer werden könnten, wenn ich älter werde“ (9 f) | 350 | 5 | **3,90** | 1,43 | 2,00 |
|  | „Ich muss darauf achten, was ich trinke“ (9g) | 353 | 5 | **3,96** | 1,53 | 3,00 |
|  | „Ich bin besorgt, eine Harnwegsinfektion zu bekommen“ (9h) | 350 | 5 | **3,50** | 1,57 | 3,00 |
|  | „Ich bin besorgt, den Katheterbeutel leeren zu können, bevor er zu voll wird“ (9i) | 344 | 4 | 4,25 | 1,24 | 1,00 |
|  | „Ich fühle mich erniedrigt durch den Katheter“ (9h) | 352 | 5 | 4,25 | 1,24 | 1,00 |
| Interpersonelle Probleme  (LQDS 4,4) | „Ich bin besorgt, meine Betreuer über die richtige Katheterpflege zu informieren“ (10a) | 344 | 5 | 4,52 | 1,00 | 0,00 |
|  | „Ich bin besorgt, Ärzte und Pflegepersonal über das Problem der „autonomen Dysreflexie“ zu informieren“ (10b) | 335 | 5 | 4,56 | 0,96 | 0,00 |
|  | „Ich bin besorgt über mögliche Konflikte mit Ärzten und Pflegepersonal in Bezug auf den Katheter (10c) | 349 | 5 | 4,55 | 0,99 | 0,00 |
|  | „Ich hatte eine schwere Zeit wegen Katheterschmerzen“ (10d) | 349 | 5 | **4,07** | 1,38 | 2,00 |
|  | „Ich bin besorgt, wie der Katheter meine Sexualität beeinflussen könnte“ (10e) | 343 | 5 | 4,57 | 1,08 | 0,00 |
|  | „Mein Katheter begrenzt die Auswahl meiner Kleidung“ (10f) | 351 | 5 | **4,08** | 1,37 | 2,00 |
| Psychosoziale Probleme  (LQDS 4,1) | „Der Katheter bewirkt, dass ich mich als kranke Person fühle“ (11a) | 353 | 5 | **4,01** | 1,39 | 2,00 |
|  | „Ich kann mein Leben wegen des Katheters weniger genießen“ (11b) | 352 | 5 | **3,92** | 1,42 | 2,00 |
|  | „Ich bin darüber frustriert, dass der Katheter mich davon abhält, zu tun, was ich mag“ (11c) | 354 | 5 | **3,99** | 1,42 | 2,00 |
|  | „Der Katheter führt bei mir zu einem Gefühl der Hilflosigkeit“ (11d) | 352 | 5 | 4,25 | 1,23 | 1,00 |
|  | „Ich habe das Gefühl, dass ich mein Zuhause nicht mehr für längere Zeit verlassen kann“ (11e) | 353 | 5 | 4,33 | 1,19 | 1,00 |
| Katheter-bezogene Lebensqualität  (LQDS 3,8) | „Ich bin besorgt über mögliche Katheterlecks“ (12a) | 351 | 5 | 3,86 | 1,45 | 2,00 |
|  | „Ich bin besorgt über einen unfreiwilligen Urinverlust“ (12b) | 351 | 5 | 3,85 | 1,45 | 2,00 |
|  | „Ich bin besorgt über möglicherweise schmerzhafte Katheterwechsel“ (12c) | 354 | 5 | 3,83 | 1,48 | 2,00 |
| Haut/  Schleimhaut  (LQDS 4,2) | „Ich bin besorgt über meine Haut im Unterbauch/Intimbereich“ (13) | 353 | 5 | **4,17** | 1,28 | 1,00 |

Tab. A1: Ergebnisse der Fragen der 5 abgefragten Domänen, n = Anzahl der Beobachtungen, na = Anzahl der fehlenden Werte, MW = Mittelwert, SD = Standardabweichung, IQR = Interquartilsabstand; **fett markiert** sind die unter dem kumulativen Gesamtpunktwert liegenden Domänen-Mittelwerte (LQDS, Lebensqualitäts-Domänen-Score)

| **Domäne** | **Frage** |  | **SPK** | **DK** | **p =** |
| --- | --- | --- | --- | --- | --- |
| Katheter-Management-Probleme | „Ich bin besorgt, wegen einer Katheterundichtigkeit nass zu werden“ (9a) | 1 | 12,1 | 12,3 | 0,209 |
|  |  | 2 | 11,6 | 14,8 |  |
|  |  | 3 | 5,3 | 11,1 |  |
|  |  | 4 | 14,7 | 11,1 |  |
|  |  | 5 | 56,3 | 50,6 |  |
|  | „Ich bin besorgt, in der Nähe eine Toilette zu finden“ (9b) | 1 | 4,8 | 4,3 | 0,806 |
|  |  | 2 | 6.4 | 6,8 |  |
|  |  | 3 | 6,4 | 9,3 |  |
|  |  | 4 | 8,0 | 9,1 |  |
|  |  | 5 | 74,5 | 69,8 |  |
|  | „Ich bin besorgt, öffentliche Toiletten zu finden, die geeignet sind, dass ich dort den Katheterbeutel leeren kann“ (9c) | 1 | 5,4 | 6,8 | 0,525 |
|  |  | 2 | 4,9 | 8,1 |  |
|  |  | 3 | 4,3 | 4,3 |  |
|  |  | 4 | 10,3 | 13,7 |  |
|  |  | 5 | 75,0 | 67,1 |  |
|  | „Ich bin besorgt über den Katheterblock“ (9d) | 1 | 6,7 | 7,3 | 0,998 |
|  |  | 2 | 7,9 | 7,3 |  |
|  |  | 3 | 5,6 | 6,0 |  |
|  |  | 4 | 11,8 | 12,6 |  |
|  |  | 5 | 68,0 | 66,9 |  |
|  | „Ich bin besorgt, dass andere den Uringeruch an mir wahrnehmen könnten“ (9e) | 1 | 10,8 | 15,0 | 0,556 |
|  |  | 2 | 9,1 | 11,9 |  |
|  |  | 3 | 12,9 | 14,4 |  |
|  |  | 4 | 14,5 | 11,9 |  |
|  |  | 5 | 52,7 | 46,9 |  |
|  | „Ich bin besorgt, dass die Katheterprobleme im Alter größer werden könnten“ (9 f) | 1 | 9,1 | 11,8 | 0,255 |
|  |  | 2 | 9,6 | 11,8 |  |
|  |  | 3 | 12,3 | 14,3 |  |
|  |  | 4 | 13,9 | 6,8 |  |
|  |  | 5 | 55,1 | 55,3 |  |
|  | „Ich muss darauf achten, was ich trinke“ (9g) | 1 | 14,2 | 12,4 | 0,542 |
|  |  | 2 | 10,5 | 14,3 |  |
|  |  | 3 | 5,8 | 9,3 |  |
|  |  | 4 | 8,9 | 8,7 |  |
|  |  | 5 | 60,5 | 55,3 |  |
|  | „Ich bin besorgt, eine Harnwegsinfektion zu bekommen“ (9h) | 1 | 19,1 | 18,8 | 0,798 |
|  |  | 2 | 9,6 | 13,3 |  |
|  |  | 3 | 14,4 | 11,2 |  |
|  |  | 4 | 14,4 | 15,0 |  |
|  |  | 5 | 42,6 | 41,9 |  |
|  | „Ich bin besorgt, den Katheterbeutel leeren zu können, bevor er zu voll wird“ (9i) | 1 | 6,1 | 5,6 | 0,473 |
|  |  | 2 | 6,6 | 6,9 |  |
|  |  | 3 | 6,1 | 9,9 |  |
|  |  | 4 | 11,0 | 11,8 |  |
|  |  | 5 | 70,2 | 62,7 |  |
|  | „Ich fühle mich erniedrigt durch den Katheter“ (9j) | 1 | 6,3 | 8,1 | 0,641 |
|  |  | 2 | 4,8 | 8,1 |  |
|  |  | 3 | 6,9 | 5,0 |  |
|  |  | 4 | 16,4 | 15,5 |  |
|  |  | 5 | 65,6 | 63,4 |  |
| Interpersonelle Probleme | „Ich bin besorgt, meine Betreuer über die richtige Katheterpflege zu informieren“ (10a) | 1 | 3,2 | 3,8 | 0,857 |
|  |  | 2 | 2,7 | 3,8 |  |
|  |  | 3 | 5,4 | 7,7 |  |
|  |  | 4 | 11,8 | 11,5 |  |
|  |  | 5 | 76,9 | 73,1 |  |
|  | „Ich bin besorgt, Ärzte und Pflegepersonal über das Problem der „autonomen Dysreflexie“ zu informieren“ (10b) | 1 | 3,3 | 2,6 | 0,320 |
|  |  | 2 | 4,9 | 1,3 |  |
|  |  | 3 | 5,5 | 6,6 |  |
|  |  | 4 | 8,2 | 11,9 |  |
|  |  | 5 | 78,0 | 77,5 |  |
|  | „Ich bin besorgt über mögliche Konflikte mit Ärzten und Pflegepersonal in Bezug auf den Katheter (10c) | 1 | 2,2 | 5,0 | 0,568 |
|  |  | 2 | 3,8 | 3,7 |  |
|  |  | 3 | 4,3 | 4,3 |  |
|  |  | 4 | 10,2 | 13,0 |  |
|  |  | 5 | 79,6 | 73,9 |  |
|  | „Ich hatte eine schwere Zeit wegen Katheterschmerzen“ (10d) | 1 | 6,9 | 11,9 | 0,157 |
|  |  | 2 | 10,1 | 9,4 |  |
|  |  | 3 | 7,4 | 7,5 |  |
|  |  | 4 | 9,0 | 15,1 |  |
|  |  | 5 | 66,5 | 56,0 |  |
|  | „Ich bin besorgt, wie der Katheter meine Sexualität beeinflussen könnte“ (10e) | 1 | 6,0 | 5,1 | 0,992 |
|  |  | 2 | 3,3 | 3,2 |  |
|  |  | 3 | 3,3 | 3,8 |  |
|  |  | 4 | 4,9 | 4,4 |  |
|  |  | 5 | 82,5 | 83,5 |  |
|  | „Mein Katheter begrenzt die Auswahl meiner Kleidung“ (10f) | 1 | 9,0 | 8,7 | 0,603 |
|  |  | 2 | 8,5 | 11,2 |  |
|  |  | 3 | 5,9 | 9,3 |  |
|  |  | 4 | 12,8 | 13,7 |  |
|  |  | 5 | 63,8 | 57,1 |  |
| Psychosoziale Probleme | „Der Katheter bewirkt, dass ich mich als kranke Person fühle“ (11a) | 1 | 10,0 | 11,2 | 0,256 |
|  |  | 2 | 4,7 | 8,7 |  |
|  |  | 3 | 15,3 | 11,8 |  |
|  |  | 4 | 7,9 | 12,4 |  |
|  |  | 5 | 62,1 | 55,9 |  |
|  | „Ich kann mein Leben wegen des Katheters weniger genießen“ (11b) | 1 | 11,1 | 13,1 | 0,841 |
|  |  | 2 | 6,3 | 8,8 |  |
|  |  | 3 | 8,9 | 10,6 |  |
|  |  | 4 | 17,4 | 16,9 |  |
|  |  | 5 | 55,8 | 50,6 |  |
|  | „Ich bin darüber frustriert, dass der Katheter mich davon abhält, zu tun, was ich mag“ (11c) | 1 | 11,5 | 9,9 | 0,476 |
|  |  | 2 | 6,3 | 11,8 |  |
|  |  | 3 | 9,4 | 9,9 |  |
|  |  | 4 | 13,1 | 11,8 |  |
|  |  | 5 | 59,7 | 56,5 |  |
|  | „Der Katheter führt bei mir zu einem Gefühl der Hilflosigkeit“ (11d) | 1 | 5,8 | 5,5 | 0,255 |
|  |  | 2 | 5,8 | 10,0 |  |
|  |  | 3 | 6,8 | 11,9 |  |
|  |  | 4 | 11,1 | 10,0 |  |
|  |  | 5 | 70,5 | 62,5 |  |
|  | „Ich habe das Gefühl, dass ich mein Zuhause nicht mehr für längere Zeit verlassen kann“ (11e) | 1 | 5,8 | 6,2 | 0,178 |
|  |  | 2 | 3,7 | 7,5 |  |
|  |  | 3 | 5,8 | 9,4 |  |
|  |  | 4 | 10,5 | 13,8 |  |
|  |  | 5 | 74,3 | 63,1 |  |
| Katheter-bezogene Lebensqualität | „Ich bin besorgt über mögliche Katheterlecks“ (12a) | 1 | 12,8 | 12,4 | 0,692 |
|  |  | 2 | 10,1 | 8,1 |  |
|  |  | 3 | 8,5 | 11,8 |  |
|  |  | 4 | 18,1 | 14,3 |  |
|  |  | 5 | 50,0 | 53,4 |  |
|  | „Ich bin besorgt über einen unfreiwilligen Urinverlust“ (12b) | 1 | 10,6 | 12,5 | 0,472 |
|  |  | 2 | 9,5 | 13,8 |  |
|  |  | 3 | 9,5 | 8,1 |  |
|  |  | 4 | 15,3 | 18,8 |  |
|  |  | 5 | 55,0 | 46,9 |  |
|  | „Ich bin besorgt über möglicherweise schmerzhafte Katheterwechsel“ (12c) | 1 | 9,9 | 14,4 | 0,384 |
|  |  | 2 | 12,5 | 12,5 |  |
|  |  | 3 | 7,3 | 11,2 |  |
|  |  | 4 | 13,0 | 13,1 |  |
|  |  | 5 | 57,3 | 48,8 |  |
| Haut/  Schleimhaut | „Ich bin besorgt über meine Haut im Unterbauch/Intimbereich“ (13) | 1 | 8,3 | 6,3 | 0,455 |
|  |  | 2 | 5,2 | 10,1 |  |
|  |  | 3 | 8,3 | 9,4 |  |
|  |  | 4 | 15,1 | 13,2 |  |
|  |  | 5 | 63,0 | 61,0 |  |

Tab. A2: Lebensqualität in Abhängigkeit von der Art des Katheters, prozentualer Anteil der kategorisierten Antworten, p-Wert des χ^2^-Unabhängigkeitstests
